# Changes in the pharyngeal airway after different orthognathic procedures for correction of class III dysplasia

**DOI:** 10.1186/s40902-022-00352-8

**Published:** 2022-06-09

**Authors:** Mohammad Saleh Khaghaninejad, Leila Khojastehpour, Hossein Danesteh, Mehdi Changizi, Farzaneh Ahrari

**Affiliations:** 1 Department of Oral and Maxillofacial Surgery, School of Dentistry, Shiraz University of Medical Sciences, Shiraz, Iran; 2 Department of Oral and Maxillofacial Radiology, School of Dentistry, Shiraz University of Medical Sciences, Shiraz, Iran; 3 Oral and Maxillofacial Surgery Resident, Department of Oral and Maxillofacial Surgery, Shiraz University of Medical Sciences, Shiraz, Iran; 4 Dental Research Center, School of Dentistry, Mashhad University of Medical Sciences, Mashhad, Iran

**Keywords:** Orthognathic surgery, Pharyngeal airway, Class III malocclusion, Oropharynx, Airway obstruction, Maxillary advancement, Mandibular setback, Bimaxillary surgery, Airway resistance, CBCT

## Abstract

**Objective:**

This study was conducted to compare changes in pharyngeal airway after different orthognathic procedures in subjects with class III deformity.

**Methods:**

The study included CBCT scans of 48 skeletal class III patients (29 females and 19 males, mean age 23.50 years) who underwent orthognathic surgery in conjunction with orthodontic treatment. The participants were divided into three groups of 16, as follows: Group 1, mandibular setback surgery; group 2, combined mandibular setback and maxillary advancement surgery; and group 3, maxillary advancement surgery. CBCT images were taken 1 day before surgery (T0), 1 day (T1), and 6 months (T2) later. The dimensions of the velopharynx, oropharynx, and hypopharynx were measured in CBCT images.

**Results:**

In all groups, there was a significant decrease in airway variables immediately after surgery, with a significant reversal 6 months later (*P* < 0.05). In subjects who underwent maxillary advancement, the airway dimensions were significantly greater at T2 than the T0 time point (*P* < 0.05), whereas in the mandibular setback and bimaxillary surgery groups, the T2 values were lower than the baseline examination (*P* < 0.05). The alterations in airway variables were significantly different between the study groups (*P* < 0.05).

**Conclusions:**

The mandibular setback procedure caused the greatest reduction in the pharyngeal airway, followed by the bimaxillary surgery and maxillary advancement groups, with the latter exhibiting an actual increase in the pharyngeal airway dimensions. It is recommended to prefer a two-jaw operation instead of a mandibular setback alone for correction of the prognathic mandible in subjects with predisposing factors to the development of sleep-disordered breathing.

## Introduction

Today, orthognathic surgery is considered the ideal option for patients with moderate to severe class III abnormality, who are affected by the esthetic, occlusal, and functional consequences of the malocclusion. Isolated mandibular setback surgery has been traditionally employed for the correction of class III dysplasia because of its greater simplicity and less associated morbidity. The posterior and inferior movement of the hyoid bone in this procedure can lead to an increase in the length of contact between the dorsum of the tongue and the soft palate and thus narrowing of the pharyngeal airway space occurs [1–3]. Airway constriction is considered a predisposing factor for obstructive sleep apnea (OSA), a life-threatening condition [4, 5]. Therefore, the popularity of isolated mandibular setback has significantly declined to the extent that today, it is performed in less than 10% of class III patients, whereas 40% undergo bimaxillary surgery [5] to reduce the risk of airway narrowing.

Numerous studies investigated the impact of orthognathic procedures on the pharyngeal airway space in patients with class III abnormality. Although the majority of studies demonstrated a significant reduction in airway volume after single-jaw mandibular setback surgery [1, 3, 6, 7], there are controversial reports about the effect of bimaxillary surgery [5, 8–12] with some studies indicating an increase [10] and others exhibiting decrease [4, 9, 12, 13] or maintenance [11, 14–16] of airway volume following a combination of maxillary advancement and mandibular setback surgery. Furthermore, most studies included small sample sizes and focused on one or two groups of patients that underwent mandibular setback or bimaxillary surgery for correction of class III dysplasia, whereas the effect of maxillary advancement on airway space has not been sufficiently compared with the two other surgical modalities at the same settings.

Another limitation in some previous studies is the use of lateral cephalograms (LCRs) for measuring the pharyngeal airway space. In LCRs, the observation and measurement of the target area are always limited to the sagittal plane, and thus, this technology cannot represent the 3-dimensional (3D) airway structure. Furthermore, the superimposition of the right and left structures and differences in magnification make a precise assessment of the images difficult. Cone-beam computed tomography provides enhanced identification and analysis of soft and hard tissues at the same time and helps achieve actual measurements by offering 3D reconstruction and multi-planar views. CBCT is now considered the standard technology for visualization of changes in the pharyngeal airway morphology following orthognathic procedures [17].

The present study was conducted to measure and compare the dimensional changes in pharyngeal airway space after different orthognathic surgeries (mandibular setback, 2-jaw surgery, and maxillary advancement) in subjects with class III abnormality through analyzing CBCT images.

## Materials and methods

### Participants

The study included CBCT scans of 48 skeletal class III patients who underwent orthognathic surgery in conjunction with orthodontic treatment. The subjects were 29 females and 19 males with a mean age of 23.50 ± 4.29 years; range 18–35 years). The inclusion criteria dictated that the subjects should be older than 18 years and require either sole maxillary or mandibular surgery or bimaxillary surgery for correction of class III discrepancy. Patients with craniofacial syndromes such as the cleft lip and palate, those with severe mandibular asymmetry as well as subjects with breathing disorders were excluded from the sample. The CBCT images that had poor diagnostic quality or did not contain the fourth cervical vertebra were also excluded. The study protocol was reviewed and approved by the ethics committee of Shiraz University of Medical Sciences, and informed consent forms were signed by all patients to allow the use of diagnostic records.

The participants were divided into three groups of 16, as follows: group 1: mandibular setback surgery, Group 2: two-jaw surgery (maxillary advancement in conjunction with mandibular setback), and Group 3: maxillary advancement surgery. The surgical procedures were contemplated by one experienced surgeon and consisted of Le Fort I osteotomy and/or bilateral sagittal split ramus osteotomy (BSSO).

### Imaging

The CBCT images were taken 1 day before surgery (T0), 1 day (T1), and 6 months (T2) after the surgical procedure. The CBCT scans were taken under standard conditions by one device (NewTom VGi EVO CBCT unit; QR SRL Co., Verona, Italy), at the following technical specifications: 75–110 kV tube voltage, 1–32 mA tube current, 24 × 19 cm field of view (to include the whole craniofacial anatomy), 0.3 mm voxel size, and 15–25 s scanning time. The patients were seated in an upright position with the Frankfort plane parallel to the ground and the teeth in maximum intercuspation. They were asked to breathe smoothly and avoid swallowing during the image acquisition. The digital image files were exported through the Digital Imaging and Communications in Medicine (DICOM) format and imported into the propriety NewTom software (NNT viewer, version 9.2) for further reconstruction and airway measurements.

### Measurements

**Fig. 1:**
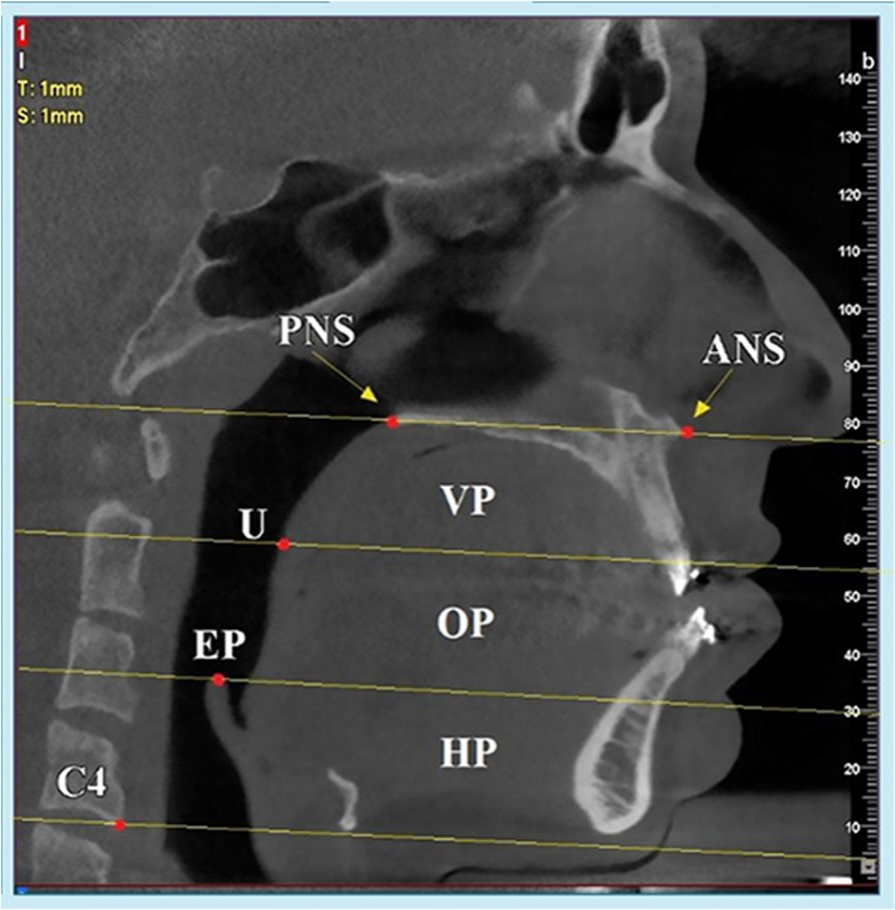
The lower pharyngeal airway was located between the palatal plane, extending to the posterior pharyngeal wall (superior limit), and a plane parallel to the palatal plane passing through vertebra C4 (inferior limit). The lower pharyngeal airway was divided into three segments including the velopharynx (VP), oropharynx (OP), and hypopharynx (HP)

The dimensional assessment of the pharyngeal airway in this study was made in the lower pharyngeal portion, which was divided into velopharynx (VP), oropharynx (OP), and hypopharynx (HP) segments (Fig. 1), as proposed by Claudino et al. [18]:

#### Velopharynx (VP)

**Fig. 2:**
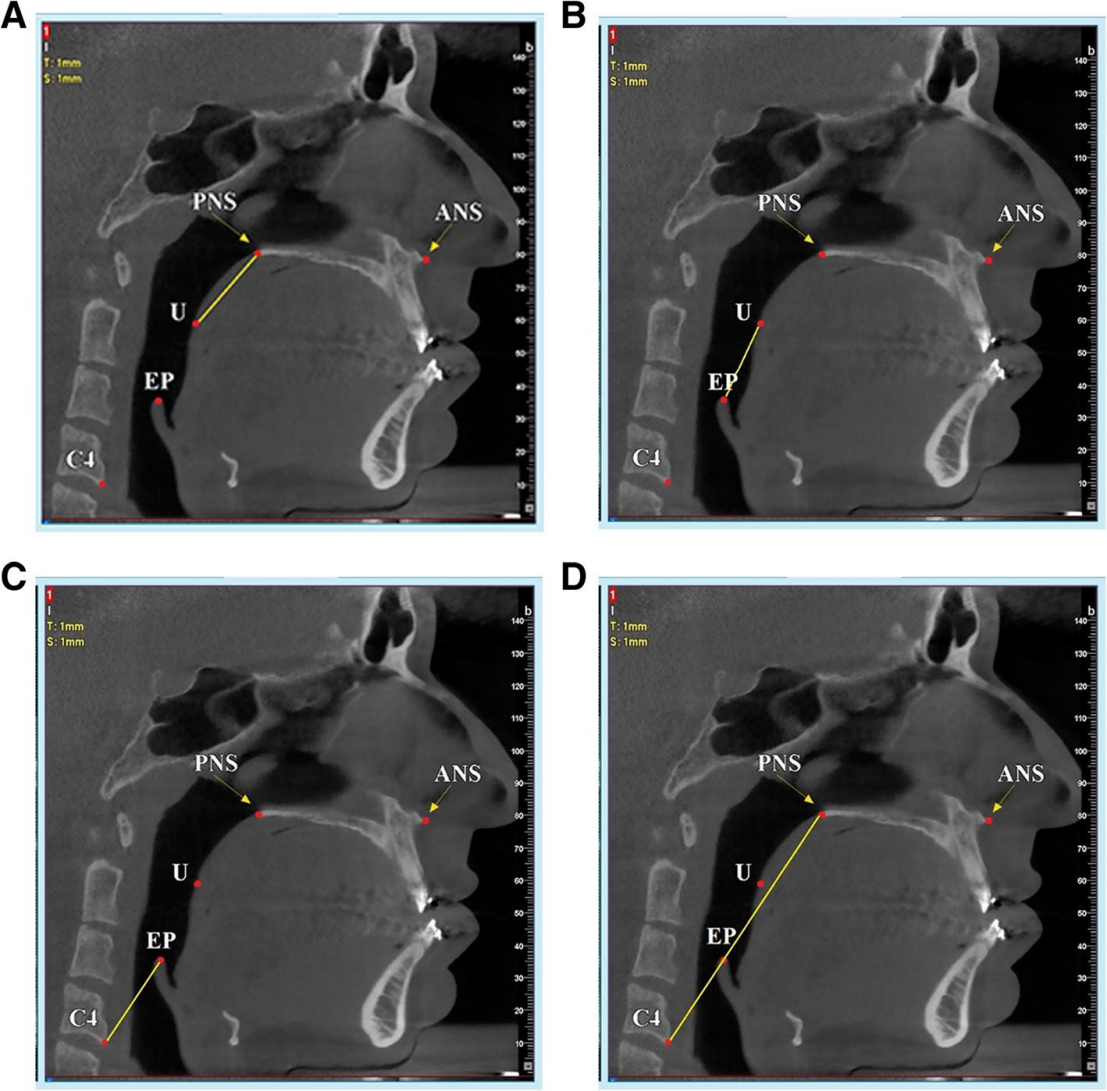
**A** The distance between the posterior nasal spine (PNS) and the uvula (U) was considered the representative of the velopharynx segment. **B** The distance between the uvula and the epiglottis (E) was considered the representative of the oropharynx segment. **C** The distance between the epiglottis and the most anterior point of the fourth cervical vertebra (C4) was considered the representative of the hypopharynx segment. **D** The distance between the PNS and C4 was considered the representative of the total pharyngeal airway

The upper boundary of the velopharynx was the palatal plane (a plane passing through the anterior nasal spine (ANS) and posterior nasal spine (PNS)), extending to the posterior pharyngeal wall. The lower limit was a plane parallel to the palatal plane that intersected the uvula (U; The most inferior point of the soft palate). In this study, the distance between the PNS and the uvula was considered the representative of the velopharynx segment (Fig. 2A).

#### Oropharynx (OP)

The upper border of the oropharynx corresponded to the lower limit of the velopharynx. The lower boundary of the oropharynx was determined by a plane parallel to the palatal plane, passing through the upper point of the epiglottis (E). The distance between the uvula and the epiglottis was considered the representative of the oropharynx segment in the present study (Fig. 2B).

#### Hypopharynx (HP)

The upper bound of the hypopharynx was the lower limit of the oropharynx, whereas its lower limit was a plane parallel to the palatal plane, which intersected the lower and most anterior point of the fourth cervical vertebra (C4). The distance between the epiglottis and the most anterior point of the fourth cervical vertebra was considered the representative of the hypopharynx segment in this study (Fig. 2C).

#### Total pharyngeal airway (TP)

The sum of the velopharynx, oropharynx, and hypopharynx was defined as the total pharyngeal airway. The superior boundary of the lower pharyngeal airway was the palatal plane, extending to the posterior pharyngeal wall, and the inferior limit was defined by a plane parallel to the palatal plane passing through the lower and most anterior point of the fourth cervical vertebra (C4). In the present study, the distance between the PNS and the most anterior point of the fourth cervical vertebra was considered the representative of the total pharyngeal airway (Fig. 2D).

### Statistical analysis

The normality of the data was confirmed by the Shapiro–Wilk test (*P* > 0.05). To detect the systemic error of the measurements, 10 CBCT scans were selected at random and measured again 1 week later by the same investigator. The paired sample *t* test revealed no significant difference between the two recordings (*P* > 0.05).

The repeated measures analysis was run to compare airway dimensions between the time intervals (T0, T1, and T2) in each of the study groups. The alterations in airway dimensions were calculated as T1 minus T0, T2 minus T1, and T2 minus T0, and the differences between groups were compared by one-way analysis of variance (ANOVA), followed by the Tukey post hoc test for pairwise comparisons. The data were analyzed by SPSS 16.0 software for Windows (SPSS Inc, Chicago, Ill, USA), and the significance level was set at *α* = 0.05.

## Results

Table 1 indicates the age and gender distribution of the participants in the study groups. The three groups were well matched in demographic data (*P* > 0.05; Table 1).

**Table 1:** The age and gender distribution of the participants in the study groups

		**Mandibular setback**	**Bimaxillary surgery**	**Maxillary advancement**	Significance
Age		24 ± 3.9	23.3 ± 4.1	23.1 ± 4.9	P=0.842
Gender	Female	10 (62.5)	10 (62.5)	9 (56.3)	P=0.917
	Male	6 (37.5)	6 (37.5)	7 (43.7)	

The Age has been shown by mean ± SD and gender by *n *(%). *SD* Standard deviation

In patients who underwent one jaw surgery, the mean amount of mandibular setback was 8.28 ± 1.68 mm and the mean amount of maxillary advancement was 8.45 ± 1.27 mm. As a result of bimaxillary surgery, the mandible moved backward 7.33 ± 0.96 mm and the maxilla moved forward 6.30 ± 1.17 mm.

### Changes in pharyngeal airway measurements throughout the experiment

Table 2 presents the mean and standard deviation (SD) of pharyngeal airway variables at T0, T1, and T2 time points in the study groups. All groups showed a marked reduction in airway dimensions immediately after surgery (T), which relapsed to some extent afterward. The T2 dimensions were lower than the T0 values in the mandibular setback and bimaxillary surgery groups, but higher in the maxillary advancement group.

**Table 2:** The mean and standard deviation (SD) for velopharyngeal (VP), oropharyngeal (OP), hypopharyngeal (HP), and total pharyngeal (TP) dimensions in the study groups at different intervals

		**To**	**T1**	**T2**	** Statistical significance**^a^
		**Mean ± SD**	**Mean ± SD**	**Mean ± SD**	
**VP**	**Mandibular setback**	10.09 ± 0.85^A^	7.56 ± 0.54^B^	8.97 ± 0.60^C^	*P*<0.001
	**Bimaxillary surgery**	9.31 ± 1.21^A^	7.04 ± 0.64^B^	8.76 ± 0.85^A^	*P*<0.001
	**Maxillary advancement**	10.87 ± 0.87^A^	9.82 ± 1.31^B^	11.56 ± 0.72^C^	*P*<0.001
**OP**	**Mandibular setback**	9.02 ± 0.96^A^	6.94 ± 0.56^B^	7.75 ± 0.63^C^	*P*<0.001
	**Bimaxillary surgery**	7.79 ± 0.61^A^	6.21 ± 0.64^B^	7.24 ± 0.70^C^	*P*<0.001
	**Maxillary advancement**	9.96 ± 0.79^A^	9.08 ± 1.43^B^	10.80 ± 0.77^C^	*P*<0.001
**HP**	**Mandibular setback**	9.31 ± 0.87^A^	7.58 ± 0.63^B^	8.27 ± 0.64^C^	*P*<0.001
	**Bimaxillary surgery**	7.79 ± 0.73^A^	6.27 ± 0.57^B^	7.38 ± 0.38^C^	*P*<0.001
	**Maxillary advancement**	10.30 ± 0.80^A^	9.26 ± 0.84^B^	10.90 ± 0.78^C^	*P*<0.001
**TP**	**Mandibular setback**	28.43 ± 1.44^A^	22.09 ± 1.29^B^	25.0 ± 1.25^C^	*P*<0.001
	**Bimaxillary surgery**	24.89 ± 1. 89^A^	19.53 ± 0.98^B^	23.38 ± 1.41^C^	*P*<0.001
	**Maxillary advancement**	31.14 ± 1.65^A^	28.17 ± 2.40^B^	33.27 ± 1.30^C^	*P*<0.001

*Abbreviations*: *SD *Standard deviation; T0, before surgery; T1, 1 day after surgery; T2, 6 months after surgery

^a^Statistically significant differences were observed at *P*<0.05. Different superscript uppercase letters denote statistical significance among the assessment intervals (horizontal) (*P* < 0.05)

**Fig. 3:**
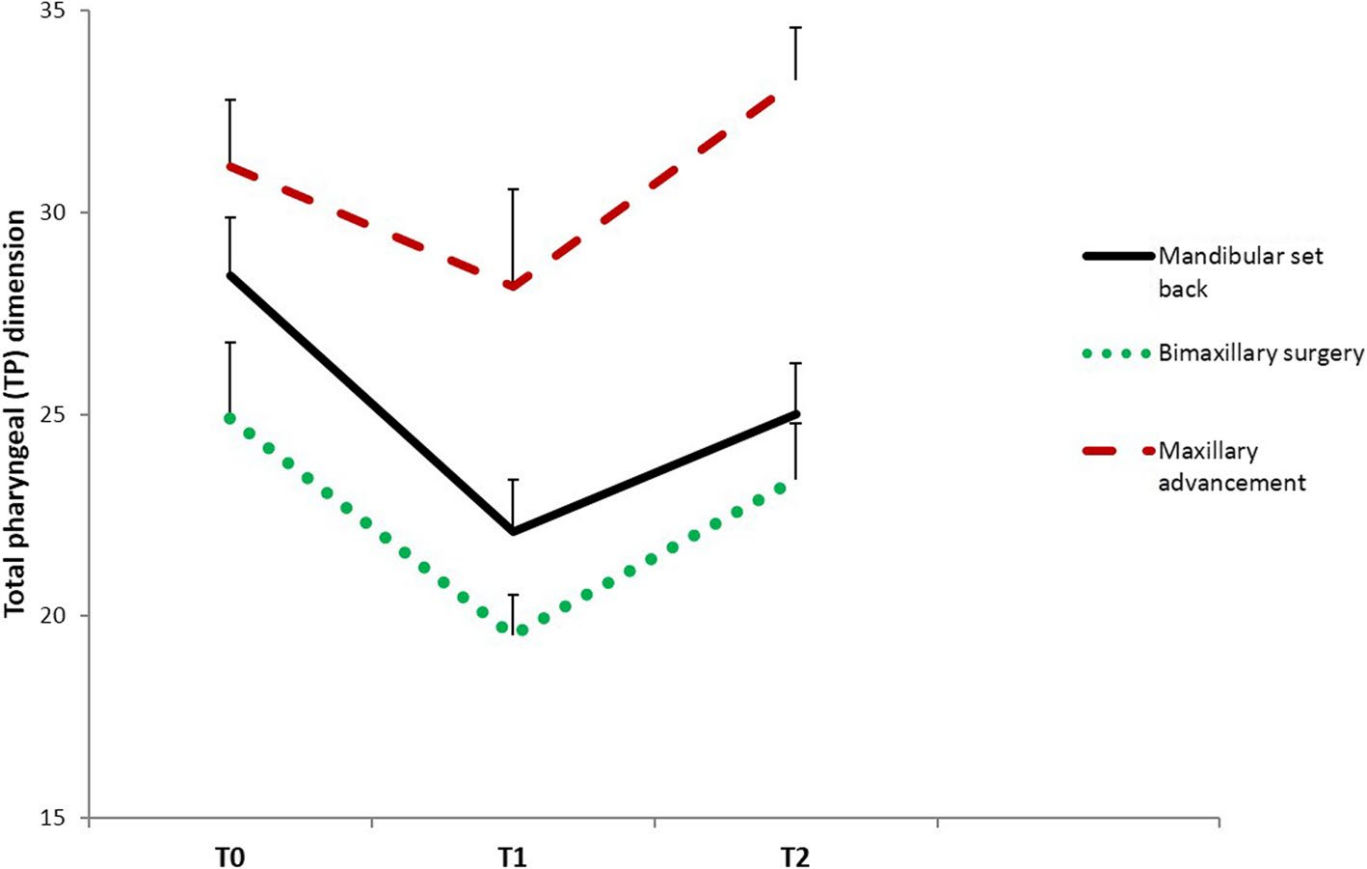
A line chart representing variations in the total pharyngeal (TP) dimension in the study groups throughout the experiment. The alterations in the TP dimension were significant in all groups throughout the experiment (*P* < 0.001)

The repeated measures analysis exhibited significant alterations in velopharyngeal (VP), oropharyngeal (OP), hypopharyngeal (HP), and total pharyngeal (TP) dimensions throughout the experiment in all the study groups (*P* < 0.001; Table 2). Pairwise comparisons by the least significant difference (LSD) test revealed that the airway measurements significantly changed from T0 to T1, T1 to T2, and T0 to T2 time points in all the study groups (*P* < 0.05; Table 2). The only exception was for the VP dimension in the bimaxillary surgery group, which showed no significant change between T0 and T2 intervals (*P* > 0.05; Table 2). Figure 3 demonstrates variations in the total pharyngeal (TP) dimension in the three groups over the study period.

### Comparisons of alterations in pharyngeal airway space between the study groups

Table 3 presents the mean and standard deviation (SD) of alterations in airway dimensions between the study groups. The positive values demonstrate an increase and the negative values represent a decrease in pharyngeal airway measurements. ANOVA revealed significant differences in T1 minus T0, T2 minus T1, and T2 minus T0 variables among the study groups for all airway dimensions (*P* < 0.05; Table 3). The only exception was the VP dimension in the T2 minus T1 variable, where all groups showed comparable alterations (*P* = 0.634; Table 3).

**Table 3:** The mean and standard deviation (SD) for alterations in velopharyngeal (VP), oropharyngeal (OP), hypopharyngeal (HP), and total pharyngeal (TP) dimensions between the study groups

		**T1-T0**	**T2-T1**	**T2-T0**
		**Mean ± SD**	**Mean ± SD**	**Mean ± SD**
**VP**	**Mandibular setback**	-2.53 ± 1.12^a^	1.40 ± 0.71	-1.12 ± 1.01^a^
	**Bimaxillary surgery**	-2.26 ± 1.17^a^	1.72 ± 0.75	-0.54 ± 1.43^a^
	**Maxillary advancement**	-1.04 ± 0.99^b^	1.73 ± 1.56	0.68 ± 1.14^b^
	Statistical significance*	P=0.001	P=0.634	*P*<0.001
**OP**	**Mandibular setback**	-2.08 ± 1.29^a^	0.81 ± 0.67^a^	-1.26 ± 1.11^a^
	**Bimaxillary surgery**	-1.57 ± 0.94^a,b^	1.02 ± 0.85^a,b^	-0.55 ± 0.77^a^
	**Maxillary advancement**	-0.87 ± 1.19^b^	1.71 ± 1.05^b^	0.84 ± 0.70^b^
	Statistical significance*	P=0.018	P=0.015	*P*<0.001
**HP**	**Mandibular setback**	-1.73 ± 0.87^a^	0.69 ± 0.49^a^	-1.04 ± 0.79^a^
	**Bimaxillary surgery**	-1.51 ± 0.55^a,b^	1.10 ± 0.53^a,b^	-0.40 ± 0.51^a^
	**Maxillary advancement**	-1.04 ± 0.67^b^	1.64 ± 1.16^b^	0.60 ± 1.00^b^
	Statistical significance*	P=0.028	P=0.005	*P*<0.001
**TP**	**Mandibular setback**	-6.34 ± 2.01^a^	2.91 ± 0.95^a^	-3.42 ± 2.16^a^
	**Bimaxillary surgery**	-5.36 ± 1.64^a^	3.85 ± 1.04^a,b^	-1.50 ± 1.83^b^
	**Maxillary advancement**	-2.97 ± 1.51^b^	5.09 ± 2.15^b^	2.12 ± 1.63^c^
	Statistical significance*	*P*<0.001	P=0.001	*P*<0.001

*Abbreviations*: *SD *Standard deviation; T0, before surgery; T1, 1 day after surgery; T2, 6 months after surgery

*Statistically significant differences were observed at *P*<0.05. Different superscript lowercase letters imply statistical significance between the study groups (vertical) (*P* < 0.05)

Pairwise comparisons indicated that the patients who underwent mandibular setback surgery exhibited the most detrimental effects on all airway dimensions, with significant differences to the maxillary advancement group in nearly all variables (*P* < 0.05; Table 3). In the bimaxillary surgery group, the changes in airway dimensions were between those of the mandibular setback and maxillary advancement groups. Tukey test demonstrated that in subjects treated with 2-jaw surgery, the alterations in VP (T1-T0, T2-T0), OP (T2-T0), HP (T2-T0), and TP (T1-T0, T2-T0) were significantly different from those of the maxillary advancement group (*P* < 0.05; Table 3), whereas other variables demonstrated comparable values to both mandibular setback and maxillary advancement procedures (*P* > 0.05; Table 3). Tukey test also revealed that for TP at the T2-T0 variable, the difference between all groups was statistically significant (*P* < 0.05), so that the mandibular setback group exhibited the greatest reduction in the pharyngeal airway, followed by the bimaxillary surgery group and maxillary advancement group, with the latter showing an actual increase in the total pharyngeal airway dimension.

## Discussion

The present study investigated the effect of various surgical procedures on the lower pharyngeal airway in patients with class III dysplasia by analyzing CBCT data. The airway measurements were performed before surgery and at 2 intervals (1 day and 6 months) after the operation. Narrowing of the pharyngeal airway following mandibular setback surgery has focused more attention in recent years, as it can lead to breathing disorders while sleeping in susceptible patients.

By analyzing the values of airway measurements in each group, it was revealed that velopharyngeal (VP), oropharyngeal (OP), hypopharyngeal (HP), and total pharyngeal (TP) dimensions changed significantly throughout the experiment. Generally, there was a significant decrease in airway variables in the study groups following orthognathic surgery, with a significant reversal 6 months later. In subjects who underwent mandibular setback surgery, the airway constriction after surgery was more pronounced and the reversal was small so that the T2 values were significantly lower than those of the pretreatment (T0) interval. In the bimaxillary surgery group, the airway dimensions at 6 months after surgery were close to the pretreatment values, but they were still lower and significantly different from the T0 measurements (except for the VP dimension in which the difference between T0 and T2 time points was not significant). The patients who underwent maxillary advancement surgery experienced an actual increase in pharyngeal airway dimensions at 6 months post-operation; thus, the T2 values were significantly greater than those of the T0 time point. The overall outcomes of this study revealed that the lower pharyngeal airway is significantly influenced by the type of surgical procedure. In patients who undergo mandibular setback surgery either alone or combined with maxillary advancement, the pharyngeal airway dimensions reduce significantly, but the degree of reduction is more pronounced in those who experience an isolated mandibular setback procedure. After maxillary advancement, an increase in airway space is expected to be observed at the 6-month interval.

In the present study, the alterations in airway dimensions were calculated between the different time points and the results were compared among the study groups. The negative values indicated a decrease and the positive values indicated an increase in airway variables. The maxillary advancement group displayed positive values for all airway dimensions at the T2-T0, corresponding to the increase in pharyngeal airway space 6 months after the operation. In the bimaxillary surgery group, the changes in airway dimensions were between those of the mandibular setback and maxillary advancement groups. The outcomes of this study exhibited that mandibular setback surgery caused the most detrimental effects on all airway dimensions, with significant differences with the maxillary advancement group in nearly all variables. The statistical analysis revealed that in the 2-jaw operation group, the alterations in some of the airway variables were comparable to both the maxillary advancement and mandibular setback groups, whereas other variables including VP (T1-T0, T2-T0), OP (T2-T0), HP (T2-T0), and TP (T1-T0, T2-T0) exhibited significant reduction as compared to the maxillary advancement group. The total pharyngeal airway at T2-T0 was the only variable that showed significant differences between all groups of the study, so the mandibular setback procedure caused the greatest reduction in the total pharyngeal airway, followed by the bimaxillary surgery and maxillary advancement groups, with the latter exhibiting an actual increase in the total pharyngeal dimension.

The significant compromise in the airway space after mandibular setback surgery may be related to the backward and downward displacement of the hyoid bone after the procedure, which moves the tongue and other muscle tissues attached to the hyoid bone in the same direction [4, 9]. The resulting change in the muscle tension contributes to the narrowing of the retrolingual and pharyngeal airway space. In contrast, maxillary advancement surgery can enlarge the pharyngeal airway and helps alleviate disordered breathing. After bimaxillary surgery, less narrowing is expected to occur in the velopharyngeal, oropharyngeal, and hypopharyngeal areas in comparison with the isolated mandibular setback process. Based on the findings of this study, it is recommended to avoid mandibular setback surgery for correction of class III deformity in subjects who have sleep-disordered breathing or show predisposing factors (such as obesity, macroglossia, short neck, large uvula, and the need to a large amount of mandibular setback) to the development of OSA syndrome [3]. In these cases, two-jaw surgery should be preferred over the mandibular setback alone for correcting the prognathic mandible, as suggested by numerous studies [5, 14, 16, 19–22].

The outcomes of this study are consistent with the results of several investigations that reported significant constriction of the pharyngeal airway after treatment of mandibular prognathism [5–7, 23]. Other studies reported worsening of sleep quality or the occurrence of sleep apnea after mandibular setback surgery in subjects with no signs or symptoms of airway obstruction before the operation [24, 25]. The present findings also corroborate the results of some studies [26–28] that indicated narrowing of the pharyngeal airway was less in subjects treated with 2-jaw orthognathic surgery than mandibular sagittal compression alone. The widening of the pharyngeal airway after maxillary advancement has also been reported in previous investigations [28–30], implying reduced airway resistance and improved breathing.

The results of the present study contradict the findings of Park et al. [31] who demonstrated that the airway capacity is maintained after mandibular setback surgery, although the structures around the mandible inevitably showed backward movement. Pereira et al. [30] exhibited no changes in the pharyngeal airway space in patients who received mandibular setback surgery. Kawakami et al. [32] indicated that the pharyngeal airway dimensions were maintained 1 month after mandibular setback surgery, although airway narrowing occurred 1 year after the operation. Gokce et al. [8] reported that 2-jaw surgery for correction of class III deformity leads to an increase in the airway volume. Saleh et al. [33] found that despite the structural modifications following maxillary advancement, the surface area and volume of the airway did not change significantly.

One of the limitations of the present study was the relatively small sample size and the short follow-up period. Only subjects with normal respiratory function were included in the study, and the effects of anatomical factors such as body size or BMI index (weight to height ratio) on the reduction of airway dimensions were not considered. Furthermore, linear measurements were employed to estimate the airway volume of different segments. Further studies with a larger sample size are warranted to elucidate the long-term effects of orthognathic surgery on upper and lower pharyngeal airway space in subjects with class III dysplasia. 

## Conclusion

According to the results from this study, the following conclusions can be drawn.

1. There were significant decreases in airway variables in the study groups immediately after orthognathic surgery, with a significant reversal 6 months later. The final airway dimensions were significantly lower than the pretreatment values in subjects who underwent mandibular setback or double jaw surgery. However, an actual increase in airway dimensions was observed at 6 months after maxillary advancement
2. The mandibular setback procedure caused the greatest reduction in the lower pharyngeal airway, followed by the bimaxillary surgery and maxillary advancement groups, with the latter exhibiting an actual increase in the airway dimensions.
3. It is recommended to prefer a two-jaw operation instead of a mandibular setback alone for correction of the prognathic mandible in subjects who show sleep-disordered breathing or display predisposing factors to the development of OSA syndrome.

## Data Availability

The datasets analyzed during the current study are available from the corresponding author on reasonable request.

## Abbreviations

VP: Velopharynx
OP: Oropharynx
HP: Hypopharynx
ANS: Anterior nasal spine
PNS: Posterior nasal spine
U: Uvula
E: Epiglottis
C4: The fourth cervical vertebra
TP: Total pharyngeal airway
